# Sarcoidosis as an Autoimmune Disease

**DOI:** 10.3389/fimmu.2019.02933

**Published:** 2020-01-08

**Authors:** Anna A. Starshinova, Anna M. Malkova, Natalia Y. Basantsova, Yulia S. Zinchenko, Igor V. Kudryavtsev, Gennadiy A. Ershov, Lidia A. Soprun, Vera A. Mayevskaya, Leonid P. Churilov, Piotr K. Yablonskiy

**Affiliations:** ^1^Laboratory of the Mosaic of Autoimmunity, St. Petersburg State University, St. Petersburg, Russia; ^2^Phthisiopulmonology Department, St. Petersburg State Research Institute of Phthisiopulmonology, St. Petersburg, Russia; ^3^Immunology Department, Institute of Experimental Medicine, St. Petersburg, Russia; ^4^Immunology Department, School of Biomedicine, Far Eastern Federal University, Vladivostok, Russia; ^5^Foreign Languages Department, St. Petersburg University of Economics, St. Petersburg, Russia

**Keywords:** sarcoidosis, autoimmune diseases, triggering factors, connective tissue diseases, HLA genotype

## Abstract

Despite the large number of performed studies, the etiology and pathogenesis of sarcoidosis still remain unknown. Most researchers allude to the possible autoimmune or immune-mediated genesis of the disease. This review attempts an integral analysis of currently available information suggesting an autoimmune genesis of sarcoidosis and is divided into four categories: the evaluation of clinical signs described both in patients with sarcoidosis and “classic” autoimmune diseases, the role of triggering factors in the development of sarcoidosis, the presence of immunogenic susceptibility in the development of the disease, and the analysis of cellular and humoral immune responses in sarcoidosis. Studying the etiology and pathogenesis of sarcoidosis will improve diagnostic procedures as well as the prognosis and patients' quality of life.

## Introduction

Sarcoidosis is a multisystem granulomatous disease of unknown origin with predominant lung involvement, variable clinical course, and no universally accepted treatment algorithm (1, 2). The disease occurs worldwide, varying in prevalence and clinical course between regions, and populations. African Americans and Northern Europeans are considered to be the most susceptible to the development of sarcoidosis with the predominance being in women, and incidence peaks from 30 to 60 years of age (2).

Recently, much attention has been paid to the study of the etiology and pathogenesis of sarcoidosis and the role of autoimmunity in its development and progression. In addition to genetic predisposition, triggers such as infection, inorganic materials, and environmental factors are likely to play a role, which leads to the possible development of an autoimmune response in the disease (2, 3).

Sarcoidosis has a wide variety of clinical phenotypes wherein many of them remind “classic” autoimmune diseases. About half of the patients have no symptoms, while in severe clinical cases, sarcoidosis can lead to a failure of the internal organ functions with the development of fibrosis and pulmonary hypertension. The most common clinical symptoms of pulmonary sarcoidosis are a dry cough, shortness of breath, and chest pain, which are described in more than 50% of patients. Up to 30% of cases include fever, unexplained weight loss, and chronic fatigue (1, 2). Extrapulmonary manifestations of the disease are described in 15–25% of cases. The most common are arthralgia, arthritis, periarthritis, acute nodular myositis, and chronic myopathy, where differential diagnosis is made with rheumatoid arthritis and other connective tissue disorders. Two main acute syndromes in sarcoidosis are described: Löfgren's syndrome manifested by nodular erythema, fever, polyarthritis, and uveitis and Heerfordt syndrome, which is described as a combination of uveitis, parotitis, fever, and facial paralysis (2, 4).

Pulmonary sarcoidosis may coexist with other autoimmune diseases, which suggests the possibility of a common pathogenesis and genetic predisposition. Most often, cases of sarcoidosis associated with Sjogren's syndrome are described, where patients have signs of both diseases, e.g., epithelioid granulomas in their internal organs as well as anti-Ro and anti-La autoantibodies as well as lacrimal and salivary gland dysfunction. Sarcoidosis and Sjogren's syndrome are both associated with the HLA-DR3 genotype and elevated levels of CD4+ lymphocytes (5, 6).

In some cases, a combination of systemic lupus erythematosus (SLE) and sarcoidosis is observed. As a rule, SLE debuts as a primary disease where over time non-caseating granulomas in the skin and lungs are detected (7). These cases suggest a common pathogenesis of both sarcoidosis and SLE regarding the similarity of some laboratory findings: In both diseases antinuclear antibodies (in sarcoidosis up to 30% of cases), a disturbance of the T and B lymphocytes ratio, and the elevation of immunoglobulin concentration are observed.

The combination of sarcoidosis with Crohn's disease, antiphospholipid syndrome, idiopathic pulmonary fibrosis, and primary biliary cirrhosis is also described (8). When sarcoidosis coexists with ankylosing spondylitis, non-caseating granulomas in the lungs, sacroiliitis, and the HLA-B27 genotype typical for spondyloarthritis are described (9).

One of the most important evidence of the autoimmune inflammation in sarcoidosis is the formation of granulomas, mainly in the lungs and the mediastinal lymph nodes as well as in the skin and liver of patients. The formation of granulomas of both infectious and autoimmune nature is based on the aggregation of hypertrophic macrophages. In infectious granulomatosis, such as tuberculosis, a pronounced activation of Th1 lymphocytes and M1 macrophages which then progress to the M2 phenotype is described. In sarcoidosis the opposite situation is observed: the M2 (anti-inflammatory) macrophage phenotype predominates in granulomas, which represents paradoxically reduced immune response. When studying the molecular mechanisms underlying the dysfunction of macrophages, there is growing evidence of the role of the mTOR pathway in granuloma formation. Linke and Wilson et al. in their studies showed that activation of rapamycin (mTOR) complex 1 (mTORC1) in macrophages in mice leads to the progression and development of granulomas. It has also been shown in people with a progressive sarcoidosis (10, 11).

After processing of environmental immunogenic antigens by mononuclear antigen-presenting cells, the antigen is presented to T-effector cells. Further activation of immune cells occurs with the help of various chemokines, such as CCL5 (for Tx1) and CCL2 (for Tx2). Recently, a lot of attention has been paid to the role of T17 lymphocytes in the pathogenesis of sarcoidosis, a subgroup of CD4+ lymphocytes expressing IL-17A, which exhibit pro-inflammatory or anti-inflammatory properties in response to the local inflammatory milieu. It has been shown that in sarcoidosis granuloma macrophages express CCR20, causing the attraction of Tx17, and the expression of IL-23 causes a significant increase in the concentration of IL-17A (11, 12). The ratio between Th17 and Treg cells probably determines the development and clinical course of sarcoidosis and can serve as a prognostic marker. Its increase indicates active sarcoidosis and correlates with the likelihood of exacerbation when steroid therapy is canceled (12).

Considering the possible autoimmune nature of sarcoidosis, it is necessary to discuss the possibilities of treatment with immunosuppressive therapy. Corticosteroids for patients with sarcoidosis are considered as the treatment of choice despite many possible contraindications and side effects (13, 14). If the corticosteroid therapy is ineffective and the disease progresses, the use of cytostatics (methotrexate, leflunomide, azathioprine) is recommended, but the doses and duration of treatment in patients with sarcoidosis require further study (15). In chronic sarcoidosis, especially in patients with extrapulmonary lesions, the use of TNF-α inhibitors (infliximab, adelemaumab, rituximab) is possible, though this question requires further study due to the immunologic abnormalities that accompany this kind of treatment and the uneven role of TNF-α in granuloma formation (16). Other biotherapy options for sarcoidosis are also being explored. The efficacy of a therapy based on inhaled vasoactive intestinal peptide (IVIP) has been shown. IVIP increases the activity of regulatory T cells and decreases the activity of effector T cells, thereby providing an immunoregulatory effect (17). There are studies on the possible beneficial effect of using IL-6 suppressors. The level of this cytokine is increased in patients with sarcoidosis and correlates with the level of CD3+ lymphocytes (tocilizumab), cytotoxic T-lymphocyte (abetacept), and IL-12/IL-23P40 and Th17 pathways, inducing the production of TNFα (ustekinumab, tildrakizumab, secukinumab) (18). The effectiveness of immunosuppressive therapy in the treatment of patients with sarcoidosis alludes to the important role of the autoimmune component in the pathogenesis of this disease.

Despite a large number of studies on sarcoidosis, its etiology is still not fully understood. To consider the possible autoimmune nature of sarcoidosis, the criteria proposed in 1993 by N.R. Rose and C. Bona can be used (19). In accordance with the classification, direct proof, indirect and circumstantial evidence are distinguished. Direct criteria include: the occurrence of the disease in a healthy individual (human or animal) with the injection of purified human antibodies, including during transplacental transfer, or *in vitro* destruction of cells carrying specific antigen. Indirect evidence includes detection of the corresponding antigen in humans or its equivalent in animals and reproduction of essential features of the disease by immunization. Circumstantial evidence includes association with other autoimmune diseases, lymphocytic infiltration, statistical association with specific HLA haplotype, and positive response to immunosuppression.

To assess sarcoidosis compliance with autoimmune disease criteria, a detailed study of risk factors, environmental, immunological, and immunogenetic triggers is necessary. Review and original articles from 1960 to 2019 were studied in the international databases (PubMed, Web of Science, SCOPUS, Elsevier, ScienceDirect). The search was performed with keywords: Sarcoidosis, autoimmune reactions, autoimmunity, antibodies, and HLA genotype.

## The Etiological Factors in the Development of Sarcoidosis: the Role of Infectious Agents as a Trigger Factor

Identifying the etiological factor in order to improve diagnosis and treatment in sarcoidosis is a very important problem (19). Numerous studies are targeting on the identification of an infectious agent that can be a trigger for sarcoidosis. Different bacteria, fungi, and viruses that can cause the formation of granulomas are described in patients with sarcoidosis (20, 21). The role of *Mycobacterium tuberculosis* and *Propionibacterium acnes* are most widely studied (22, 23). Particular attention is paid to the infection of *M. tuberculosis*, its role in the development of sarcoidosis since the 1960s has been observed by clinicians. One of the first papers tackling the effect of *M. tuberculosis* on the development of sarcoidosis is the study of clinical cases in which the association of a previous tuberculosis infection with the subsequent development of sarcoidosis is shown. According to the data described, treatment with anti-TB drugs was not effective, while the use of corticosteroids led to a decrease in granulomas and the reduction of clinical symptoms (24).

With the development of molecular genetic methods, it became possible to identify specific markers of *M. tuberculosis*. Since the 1980s the presence of mycobacterial genetic material in patients with sarcoidosis was studied, according to which *M. tuberculosis* DNA or RNA was detected in 20–50% of cases (22). Serological methods for confirming the association of mycobacterial infection and the development of sarcoidosis originate from the 1990s with studies of the cross-reactions of bacterial antigens with patient serum. Antibodies to mycobacterial proteins p36, heat shock proteins hsp 65 and hsp70 were found in sarcoidosis patients (25). Studies by Ang et al. have shown a cross-reaction of mycobacterial antigens with cytoskeleton proteins of Schaumann bodies (tubulin, desmin, vimentin) (26). Activation of a cytotoxic cellular response in peripheral mononuclear cells of patients with sarcoidosis in response to *M. tuberculosis* specific antigens has been shown during incubation of mononuclear cells with ESAT-6 and KATG proteins (24, 27).

The possible evidence of the role of *M. tuberculosis* in the pathogenesis of sarcoidosis might be shown with the studies on animal models. The development of sarcoid granulomas was shown by Chen et al. in an experiment with Lewis rats and C57BL/6 mice. The formation of granulomas was described after the injection of cell lysates and recombinant catalase of *M. tuberculosis* (28). Later, Swaisgood et al. investigated the role of mycobacterial superoxide dismutase A in C57BL/6 mice where the development of granulomas and increased concentrations of CD4+ cells, IL-2, and IFN-γ in bronoalveolar lavage were shown (29).

Given the possible role of *P. acnes*, Werner et al. performed an experiment on the C57BL/6 mice, where the live strain of the microorganism isolated from the respiratory tract of a sarcoidosis patients was injected. After several days, the formation of granulomas and an increase in IFN-γ producing CD4+ cells were detected in animal tissues, while the size of granulomas was more significant than in experiments with heat-killed strains (30). The main issue of these models is the question of specificity and chronicity of granuloma formation. Because the results of these studies were contradictory, and there is no consensus among researchers about the exact relationship between the role of the infectious agent in the development of sarcoid granulomas, further studies are required (31).

The role of fungi in the development of sarcoidosis was studied by examining office staff working in old buildings. A tendency for an increase in the incidence of sarcoidosis and the presence of fungi in the dust was noted (32, 33). There are no studies showing the direct effect of infectious agents on the development of granulomas in humans. An important counterargument is also the absence of microorganisms in granulomas.

## The Role of Inorganic Dust and Other Exogenous Triggers in Sarcoidosis

Among the chemicals which may be a triggering factor in patients with sarcoidosis, silicone holds a special place (34). This material, being considered biologically inert, is widely used in breast implants, artificial joints, shunts for patients with hydrocephalus, and catheters. It has been shown that in predisposed patients, at the site of its administration, giant cell granulomas containing silicone particles may form, and the specific clinical symptoms may be observed (34, 35). When silicone implants are removed, the described changes regress in 60–80% of cases (34).

A recent study by A. Watad comparing 24,651 patients with silicone implants and 98,604 patients without foreign bodies showed an increased risk of autoimmune diseases (OR 1.21, 95% CI 1.17–1.26), the most pronounced associations being with sarcoidosis, Sjogren's syndrome, and systemic scleroderma after silicone implantation (34). These cases demonstrate the role of silicone as an adjuvant in the progression of sarcoidosis with the development of both focal (the appearance of sarcoid granulomas in the skin at the site of silicone mammoplasty or subcutaneous injections), and most importantly, systemic manifestations of sarcoidosis (8, 35).

One of the possible triggering factors that increases the incidence of sarcoidosis is inorganic dust. In epidemiological studies, the mechanism and specific components of dust causing granuloma formation were not demonstrated, however, an increase in the incidence of sarcoidosis after the collapse of the World Trade Center on September 11, 2001 was recorded (36, 37). When studying the composition of inorganic dust that appeared after the collapse of the Center, it was found that it consisted mainly of concrete components, including gypsum, anhydrite (anhydrous calcium sulfate), and fiberglass. The obtained data suggested the effect of inorganic triggers of sarcoidosis (silicates, talc, and others), which were described earlier in a number of clinical cases as causal factors of sarcoidosis development (35, 37).

There is some evidence in scientific literature of the effect of printer or copier toner ink on the development of sarcoidosis. For example, Armbruster et al. described a 39-year-old non-smoker who, after 18 months working at a newspaper agency, had intrathoracic lymphadenopathy and disseminated changes in the lungs (38). Histological biopsy examination of the lungs and lymph nodes revealed epithelioid cell granulomas without caseous necrosis; an additional examination of the obtained tissues revealed toner dust microparticles. Although granulomatous pneumonitis with mediastinum lymphadenopathy was diagnosed due to copy machine emissions, the patient most likely suffered from pulmonary sarcoidosis of the lungs and intrathoracic lymph nodes. Given the likely multifactorial nature of sarcoidosis and the role of various adjuvants acting as triggers for the development of the disease, the impact of such a multicomponent substance as ink in printer toner or copier, especially with long-term and direct use, can be considered as one of the adjuvants for sarcoidosis. The toner particles diameter is from 2 to 10 μ, when inhaled, they are deposited mainly in the tracheobronchial and alveolar regions. Toners are composed of thermoplastic polymer particles, usually styrene-acrylate copolymer, which are fixed on the paper by melting. Black toners contain carbon black or iron oxide as pigments, and organic pigments are included in colored toners (38). In addition, toners contain additives such as wax and silicate, whose “positive” effect on the development of sarcoidosis has been considered many times before.

Thus, numerous studies including epidemiological analysis, *in vitro* experiments, experiments on animal models, and clinical observations have shown the role of environmental factors (silicates, insecticides, silicone, etc.), infectious agents (*Propionibacterium acnes, M. tuberculosis*, and fungi) in the development of sarcoidosis. These factors can play an important role in the development of the disease (39).

## The Role of Vimentin as an Autoantigen in Sarcoidosis

The role of vimentin—a peptide presented in the cells of the connective tissue, which is involved in intercellular interactions and functioning of the immune system, has been known for a long time in the development of autoimmune diseases. Recent studies suggest that the mesenchymal protein vimentin, may be an autoantigen for sarcoidosis. The presence of autoantibodies to this protein is described in the pathogenesis of rheumatoid arthritis, systemic lupus erythematosus, and many other systemic autoimmune diseases (40, 41).

The first mention of vimentin in sarcoidosis was made when studying the asteroid bodies of the giant multinucleated cells in granulomas. The researchers discovered that asteroid bodies consisted of vimentin filaments and were the result of an aggregation of filamentous and microtubular systems of the centrospheres, which might be due to local liquid shifts, and sol-gel transformations. The authors suggested that such changes in the structure of giant cells could lead to their dysfunction and exhaustion, which might play an important role in granuloma development (42).

A possible relationship between vimentin and sarcoidosis development was shown in the study on the cross-reaction of antimycobacterial antibodies with Schaumann body proteins in giant sarcoid granuloma cells. Granulomas showed the highest intensity for lysosomal proteins of muramidase (muramidase proteins) and CD68, which was different for some cytoskeleton proteins (tubulin, desmin, vimentin) (26).

Despite the fact of elevated levels of vimentin in granulomas, for a long time there was no evidence of an autoimmune response against this protein. Since 2007, the Wahlström group has studied cellular and humoral response in sarcoidosis and detected specific T-cells and antibodies to vimentin in representatives of the HLA-DR-B1^*^0301 genotype (43). The role of vimentin in the pathogenesis of sarcoidosis was shown in Eberhardt's studies of T-cell response in the incubation of mononuclear cells with Kveim and vimentin reagent (44).

Despite the data obtained, it is difficult to state whether vimentin is an autoantigen in sarcoidosis. To confirm this hypothesis it is necessary to identify autoantibodies to vimentin, establish the cytotoxicity of vimentin-specific T and B cells against tissues, and also obtain a model of the disease in animals by injection of vimentin protein. This requires further study.

## Immunogenetics of Sarcoidosis: a Brief Synopsis of Autoimmune Disorders in Sarcoidosis

The immunogenetic features of autoimmune pathologies include their relationship with the HLA genotypes. The HLA genotypes are associated with many autoimmune diseases, which enabled us to use genotyping of their loci to determine susceptibility to certain diseases, the nature of the disease course, and the effectiveness of treatment (45).

The MHC class I molecules (A, B, C) are presented on the membrane surfaces of all cell types, with the exception of trophoblast cells, and present antigens localized in the cells' cytosol of T lymphocytes. The MHC class II molecules (DQ, DR, DP) are predominantly expressed in the antigen-presenting cells, which represent antigens absorbed from the extracellular space by endocytosis. Presentation of the body's own antigens in association with MHC I or MHC II and their subsequent recognition by autoreactive cytotoxic T lymphocytes or T-helper cells, respectively, is a key mechanism for triggering autoimmune reactions (46).

Many characteristic HLA genes were found in sarcoidosis that encoded both molecules of the class I (some isoforms of HLA-A and –B) and class II (some of the isoforms of HLA are DPB1, -DQB1, -DRB1, -DRB3). Specific HLA genotypes have been identified for the various forms of the disease, the spectrum of the affected organs, as well as ethnic characteristics. For example, it was shown that carriers of the HLA-DRB1^*^04/^*^15 alleles are more likely to develop cardiovascular sarcoidosis, and the HLA-DRB1^*^04 alleles are more likely to develop uveitis (47). The HLA-DRB1^*^03 and HLA-DQB1^*^0201 alleles are associated with the development of Löfgren's syndrome, while the HLA-DRB1^*^15 and HLA-DQB1^*^0601 alleles are associated with chronic sarcoidosis, which is reflected in more detail in Table 1.

**Table 1:** Genotypes affecting the development and severity of sarcoidosis (41–45).

**References**	**HLA genotypes**	**Discovered relationship with various sarcoidosis manifestations**
Bogunia-Kubik et al. (48)	HLA-DRB1^*^03	Favorable prognosis, acute course
	DRB1^*^15	Chronic course
Planck et al. (49)	DQB1^*^0201	Favorable prognosis, acute course
	DQB1^*^0602	Chronic course
Foley et al. (50)Kishore and Petrek (39, 51)Fingerlin et al. (52)Moller et al. (20)	HLA-DRB1^*^01/04 HLA-B08 HLA-DRB1^*^03:01	Löfgren's syndrome, acute course
	HLA-DRB1^*^12/14 HLA-DRB1^*^14:01 HLA-DRB1^*^04/15	Chronic course Extrapulmonary lesions
Grunewald et al. (47)	DRB1^*^07 DRB1^*^14 DRB1^*^15	Chronic course, poor prognosis
	DRB1^*^01 DRB1^*^03	Löfgren's syndrome, good prognosis

Among different ethnic groups most often affected by sarcoidosis—African Americans and Europeans—various HLA genotypes were found. The presence of HLA-DRB1^*^11:01 increased the risk of disease in both ethnic groups, whereas HLA-DRB1^*^12: 01/15: 03 was more common for African Americans, HLA-DRB1^*^15: 01 / 04:01—for the Caucasian race (50, 53). At the same time, the HLA-DRB1^*^03: 01 genotype for Europeans is a predisposing factor for the development of sarcoidosis, while for African Americans this genotype is of a protective value (48).

The class III of HLA genes was shown to be associated with sarcoidosis of the following genes: BTNL2, C4, C6orf10, HSPA1L, LTA, NOTCH4, TAP2, TNF, and VEGF. These genes are involved in many cellular processes and play a large role in all stages of inflammation. For example, the products of the NOTCH4 gene are Notch family proteins that control cell division during growth, differentiation, apoptosis and regulate T-cell immune response. It was found that the NOTCH4 gene is associated with the development of other autoimmune diseases (systemic sclerosis, neonatal lupus, multiple sclerosis). The role of this gene in the pathogenesis of sarcoidosis is not yet known, but its relationship with sarcoidosis among African Americans, and Europeans has been shown (54).

The described immunological and immunogenetic disorders do not arise independently, but under the influence of certain triggering factors that activate and trigger the pathogenesis mechanism of the autoimmune response. Currently, this relationship is described in many studies.

## Immunopathogenesis of Sarcoidosis: Characteristic of Autoimmune Diseases in Patients With Sarcoidosis

The main role in the pathogenesis of autoimmune diseases is played by T-cell immunity represented by the 1, 2, 17 Th subtype of T helpers, regulatory T-cells, and also T-killers. It is believed that disturbance of the Th17 ratio stimulates the T regulatory cells, which play a role as suppressors of immune responses, and can lead to autoimmune inflammation characterized by the presence of autoantigen-specific T and B lymphocytes producing autoantibodies (55).

In various studies on sarcoidosis, disturbances were found in T-cell and B-cell immune responses. It is believed that the triggering factor for inflammation development in sarcoidosis is the contact of the foreign antigen with antigen-presenting cells leading to activation of T and B lymphocytes, which migrate to the inflammatory foci. It is observed, that due to the long persistence of the antigen, macrophages undergo epithelioid differentiation, lymphocytes constantly migrate to the center, thus, granulomas are formed (56, 57).

Considering that lymphocytes play the main role in granuloma formation, it can be said that the disturbance of cellular and humoral immune response is observed in sarcoidosis.

## Cellular Immune Response

As has been noted above, a distinctive feature of sarcoidosis is the formation of non-caseous granulomas, which include various immune competent cells. Thus, in the central part of such granulomas, macrophages or multinucleated giant cells and CD3+ CD4+ T-helpers predominate as a result of their fusion, whereas cytotoxic CD3+ CD8+ T-lymphocytes, regulatory T-cells, fibroblasts, and B-lymphocytes predominate in the peripheral part of the granulomas (56, 57). The leading role of tissue macrophages in the formation of granulomas in sarcoidosis was brought to light in the so-called “Th1-paradigm” of immunopathogenesis of this disease, which was based on the leading role of IFNγ and T-helper type 1 as the main producers of this cytokine indispensable for the activation of macrophages to induce and maintain inflammatory processes in tissues during chronic activation of macrophages (58).

This circumstance allowed us to treat sarcoidosis primarily as an infectious disease. However, the description of a fundamentally new population of T-helper cells—T-helper 17 (Th17) as well as its separate highly specialized subtype—Th1/Th17 or Th17.1 cells capable of producing both IFNγ and IL-17A at the same time, suggests the autoimmune nature of the disease (58, 59). Furthermore, in a number of studies, an increase in the level of Th17.1 and the proinflammatory cytokines produced by them in the peripheral blood and the composition of the bronchoalveolar lavage (BAL) fluid of patients with sarcoidosis was detected. An increase in the level of these cells, both at the systemic level (in the peripheral blood) and directly in the focus of the inflammatory reaction and damage to surrounding tissues was observed in a wide range of other systemic—for example, Sjögren syndrome and rheumatoid arthritis, and organ-specific— for example, type 1 diabetes mellitus and Crohn's disease (59).

In patients with sarcoidosis, an increase in Th17 was found in the peripheral blood and BAL, while the number of T regulatory cells in BAL was reduced and increased in the peripheral blood (60). Also, in many studies there was a decrease in the level of transcription factor T-regs FOXP3 in BAL, which indicates a decrease in cell function. Since Th17 contributes to fibrosis, and T-regs reveal anti-proliferative activity, the variability of their ratios may explain an increased proliferation of connective tissue in the affected organ as well as being one of the confirming factors of the autoimmune nature of sarcoidosis (61, 62).

Cytotoxic T-lymphocytes, along with Th, effectively produce key pro-inflammatory cytokines (TNFα and IFNγ), ensuring their high concentration at the sites of granuloma formation, which activates macrophages and attracts additional cells from the circulation (63). At present, the involvement of CD3+ CD8+ lymphocytes is shown in the pathogenesis of various forms of arthritis, multiple sclerosis, type 1 diabetes mellitus, and many other autoimmune diseases (64, 65). However, the role of CD8+ T-lymphocytes in granuloma formation in sarcoidosis remains poorly studied, which does not prevent us from considering this cell population as a promising therapeutic target in the treatment of sarcoidosis.

One of the main criteria for autoimmune disease is the presence of auto-antigen-specific T-lymphocytes. In 2012, Ahmadzai et al. described in sarcoidosis the activation of the peripheral mononuclears followed by the release of various cytokines after stimulation with autoantigens (vimentin, lysyl-tRNA synthetase) (66). Later, in the Grunewald study, the presence of specific type 1 CD4+ T-helpers of the 1st type with Vα2.3/Vβ22 receptors interacting with HLA-DRB1^*^03 proteins was detected (67). When studying the receptors, the protein structure was calculated, which coincides with the vimentin structure represented by antigen-presenting cells using HLA-DRB1^*^03 (68).

## Humoral Immunity: Antibody-Mediated Reactions in Sarcoidosis

The earliest studies on the immunological characteristics of sarcoidosis have shown that patients with sarcoidosis have a significantly higher number of B-cell activating factor (BAFF) and, correspondingly, Ig-producing B-cells in the lung tissues, while there are various ratios of B-cell types (69, 70). In BAL of patients with sarcoidosis, there was a decrease in the number of memory B-cells, an increase in the number of B-regulatory cells producing IL-10, and hypergammaglobulinemia correlating with an increase of immunoglobulins in the peripheral blood (71). The use of anti-B-cell therapy (antibodies against CD20, rituximab) in the treatment of sarcoidosis resulted in clinical improvement, which suggests the participation of both “naive” B-cells and memory B-cells in the pathogenesis of the disease (72, 73). Hypergammaglobulinemia has been shown in several studies, but the association with the severity of the disease was not detected (74). Although some authors consider that patients with sarcoidosis have normal levels of serum immunoglobulins, but applying histological methods in the analysis of the subpopulation composition of cells forming granulomas, the accumulation of B cells in the lungs' lesions was shown (70).

The earliest data on autoantibodies in sarcoidosis were based on the results obtained from the patients diagnosed with sarcoidosis and various chronic liver diseases, in whom antimitochondrial antibodies were identified. Similar results were obtained in 2005 when examining patients with sarcoidosis and primary biliary cirrhosis. The authors find it difficult to name the underlying cause of an autoimmune response because the presence of antimitochondrial antibodies is characteristic of autoimmune liver damage (75–77).

Since sarcoidosis combines with other autoimmune diseases, the Weinberg group analyzed the presence of anti-nuclear antibodies and autoantibodies to double-stranded DNA to determine whether these markers could be used as predictors of disease development, such as systemic lupus erythematosus. However, antinuclear antibodies were detected in only one third of the patients, whereas antibodies to double-stranded DNA were in two patients, and therefore, these serological markers are not diagnostically significant (78).

In 2014, the S. Kobak group began studying autoantibodies specific for rheumatoid arthritis in patients with sarcoidosis. Of the 42 patients with sarcoidosis, only seven had a higher concentration of the rheumatoid factor, two of them had antibodies to citrullinated cyclic peptides. The anti-nuclear antibodies were detected in 12 patients. However, due to the low specificity of the detected antibodies, these serological indicators cannot be used as biomarkers of sarcoidosis (79, 80).

In 2018, Johan Grunewald's group showed the possible association of vimentin as an autoantigen in the development of sarcoidosis and published an article about the presence of autoantibodies to vimentin in serum and bronchoalveolar fluid of patients with sarcoidosis. At the same time, association with the HLA-DRB1^*^03 genotype was revealed (81).

Table 2 presents studies dealing with various types of autoantibodies detected in patients with sarcoidosis.

**Table 2:** Detection of various autoantibodies in sarcoidosis (74–80).

**References**	**Study, Number of patients with sarcoidosis**	**Number of patients with changes**	**Detected antibodies**
Maddrey et al. (75)	20 patients with sarcoidosis and chronic liver damage	1/20 5.0%	Antimitochondrial antibodies
Fagan et al. (76)	4 patients with sarcoidosis and liver damage	4/4	
Stanca et al. (77)	Clinical case: patient with sarcoidosis and primary biliary cirrhosis	1/1	
Weinberg et al. (78)	34 patients with sarcoidosis	10/34 29.4%	Antinuclear antibodies
		2/34 5.8%	Antibodies to double stranded DNA
Kobak et al. (9, 79, 80)	42 patients with sarcoidosis	2/42 4.7%	Antibodies to citrullinated cyclic peptide
		7/42 16.7%	Rheumatoid factor
Total six studies	101 patients with sarcoidosis	27/101 (26.7%)	-

According to the data presented in Table 2, the various autoantibodies were evaluated in patients with sarcoidosis in 26.7% of cases with variability from 4.7 to 29.5%.

## The Role of Innate Immunity and Auto-Inflammation in the Pathogenesis of Sarcoidosis

According to the proposed model of sarcoidosis, the antigen-presenting cells (macrophages, dendritic cells and epithelial cells) are the first in the cascade of inflammatory reactions. It is believed that with prolonged contact of foreign antigens with the toll-like receptors of antigen-presenting cells, macrophage activation and epithelioid differentiation occur. Macrophages begin to synthesize pro-inflammatory cytokines such as nuclear factor (NF)-kB, TNF-a, IL-1, which leads to the activation of the acquired immune system. B-lymphocytes and T-lymphocytes actively migrate to the focus of inflammation. T-cells undergo differentiation into T-helpers of the 1st, 2nd, 17th type, and regulatory T-cells, depending on secreted cytokines. The inability to quickly and efficiently eliminate a foreign antigen leads to the formation of granulomas (20, 21). However, unlike the typical granulomatous process, macrophages in sarcoidosis begin to differentiate into M2 type, which have anti-inflammatory properties and contribute to the chronicity of the process and fibrosis due to the production of profibrotic chemokine CCL18 (82).

Autoinflammatory diseases are characterized by chronic systemic inflammation caused by activation of innate immunity (neutrophils, macrophages) and manifested in lesions mainly of the musculoskeletal system, skin, and mucous membranes (83). This group includes early-onset sarcoidosis, which occurs in children and is characterized by the formation of non-caseating granulomas in various organs. Most often, symmetric arthritis, dermatitis, uveitis and neuropathies can be found. However, with this type of sarcoidosis, the etiology of the disease is already identified and described as the presence of mutations in the CARD15 [caspase activation and recruitment domain (CARD) family, member 15] or NOD2 (nucleotide-binding oligomerization domain containing 2) gene (84).

As shown above, the development of sarcoidosis is associated with a large number of genes, in particular with class II HLA genes. Moreover, with sarcoidosis, autospecific T and B cells that produce various autoantibodies are found. These facts do not allow to suggest an exclusively auto-inflammatory theory of sarcoidosis. Perhaps sarcoidosis begins as an auto-inflammatory disease in which the activation of adaptive immunity occurs with the appearance of autospecific T and B cells that produce autoantibodies to a specific antigen (85).

## Conclusion

According to the criteria for autoimmune diseases proposed by Rose and Bona (19), in sarcoidosis only circumstantial evidence of autoimmune inflammation is described:

- the presence of various autoantibodies in the blood of patients;
- association with other autoimmune diseases (SLE, Sjogren's syndrome);
- association with the HLA genotype (HLA-DRB1, HLA-DQB1);
- lymphocytic organ infiltration (the presence of sarcoid granulomas);
- response to immunosuppressive therapy.

At present, there is lack of evidence on the possible autoantigen in sarcoidosis, which induces the formation of sarcoid granulomas, antibodies, and T-specific lymphocytes in animal models to confirm the autoimmune origin of sarcoidosis. Vimentin appears to be the most likely antigen, against which the Grunewald group has detected both antibodies and vimentin-specific T cells.

The data analyzed in the review suggest the presence of autoimmune/inflammatory syndrome induced by adjuvants described in 2011 by Shoenfeld and Agmon-Levin (86). The syndrome implies various autoimmune conditions that occur under the influence of various triggering factors in individuals with a certain immunogenetic predisposition, which trigger immunological reactions characteristic of autoimmune diseases.

The described major (clinical) and minor (immunogenetic) ASIA criteria enable us to assume the autoimmune nature of inflammation in sarcoidosis. Analyzing the proposed algorithms to detect autoimmune diseases, a unified approach to assess the possible autoimmune origin of sarcoidosis can be proposed: Identification of trigger factors affecting the development of sarcoidosis; evaluation of immunogenetic susceptibility to the development of the disease; analysis of the immunological features in sarcoidosis; analysis of the clinical features of autoimmune diseases.

The analysis of the current data does not exclude the auto-inflammatory nature of the occurrence and development of sarcoidosis. Thus, setting the final point in the etiology and pathogenesis of the disease is not yet possible.

## Author Contributions

AS: main author. AM, NB, YZ, IK, GE, LS, and LC: analysis of literature. VM: text translation. PY: coordinator of the project.

### Conflict of Interest

The authors declare that the research was conducted in the absence of any commercial or financial relationships that could be construed as a potential conflict of interest.
